# Synergistic regulation mechanism of N in sugarcane-soil systems through ^15^N tracer and ecological stoichiometry

**DOI:** 10.3389/fpls.2026.1809173

**Published:** 2026-04-27

**Authors:** Ning Zhong, Jinsheng Huang, Qiuliang Cai

**Affiliations:** 1 Fujian Province Key Laboratory of Modern Analytical Science and Separation Technology, College of Chemistry, Chemical Engineering & Environmental Science, Minnan Normal University, Zhangzhou, China; 2 Key Laboratory of Urban Environment and Health, Institute of Urban Environment, Chinese Academy of Sciences, Xiamen, China; 3 Agricultural Resources and Environmental Research Institute, Guangxi Academy of Agricultural Sciences, Nanning, Guangxi, China; 4 Guangxi Key Laboratory of Biology for Mango, Agriculture and Food Engineering College, Baise University, Industrial College of Subtropical Characteristic Agriculture, Baise, Guangxi, China

**Keywords:** N management, N uptake and distribution, nutrient stoichiometry, soil quality, synergistic regulation

## Abstract

Sugarcane is a vital sugar crop in China. Low nitrogen use efficiency (NUE) is a key constraint to the sustainable development of the industry. To comprehensively evaluate the regulatory effects of N management on the sugarcane-soil system and its nutrient coordination, a controlled pot experiment was carried out using the Guitang 46 sugarcane variety in Baise, Guangxi. Six N treatment strategies were implemented: no N input (control), split applications at low, medium, and high levels, and one-time applications at medium and high levels. A combination of conventional agronomic measurements, ^15^N isotope tracing, and ecological stoichiometry analysis was employed to investigate the influence of these treatments on (i) sugarcane growth (biomass accumulation, N uptake and allocation), (ii) soil properties (physicochemical characteristics), and (iii) system-level nutrient balance (C:N:P stoichiometric relationships within the plant–soil system).The findings revealed that the medium one-time N application delivered the most favorable outcomes. Specifically, it enhanced stem dry matter production (327.75 g) and total plant N uptake (4.79 g/plant) by 42.87% and 62.37%, respectively, compared to the control. Furthermore, it achieved a ^15^N recovery rate of 40.78% and minimized N losses to just 20.66%. This treatment also contributed to an optimal stoichiometric balance—reflected in a stem C:N ratio of 28.5, a new leaf N:P ratio of 14.2, and a soil C:N ratio of 18.7, closely aligning with the ideal microbial decomposition threshold. Additionally, this approach significantly improved soil organic matter (OM, 63.97 g/kg), increased available N, and sustained stable invertase and urease enzyme activity. Taken together, these results demonstrate that a medium one-time N application simultaneously promotes high sugarcane yield, efficient N utilization, enhanced soil quality, and nutrient balance within the crop-soil system. These findings provide robust scientific support for reducing fertilizer input while maintaining productivity in sugarcane cultivation.

## 1 Introduction

Sugarcane (*Saccharum officinarum* L.) serves as China’s most important sugar-producing cash crop, accounting for over 92% of the country’s total sugar output. Among its growing regions, Guangxi Province stands out, contributing more than 70% of the overall yield from major regional cash crops. This underscores the strategic importance of sugarcane cultivation in ensuring national sugar security and promoting regional agricultural economic growth (Wang and Cai, 2024).

N is widely recognized as a critical limiting nutrient for sugarcane development and yield formation. The efficiency with which it is managed directly influences both the economic returns and the ecological sustainability of sugarcane production systems. However, a major challenge persists: the current N use efficiency (NUE) in China averages only 20%~30%, which is significantly lower than that in agriculturally advanced countries such as Australia (Bakker, 1999). Over-application of N not only inflates production costs but also degrades soil structure, disrupts nutrient balance, and exacerbates N (NO_3_^−^) leaching and non-point source pollution—factors that collectively hinder the sustainable development of the sugarcane sector (Conen et al., 2008; Li et al., 2024).

In response, the development of N management strategies that can concurrently improve crop yield, nutrient efficiency, and soil quality has become a pressing issue in the field of sugarcane agronomy and soil ecology. Recent research has explored how N inputs affect the sugarcane-soil system, revealing that N plays a regulatory role in both plant physiological traits (e.g., height and biomass/plant) and soil nutrient status (e.g., organic matter (OM), N, P, and K) (Wang and Cai, 2024). Additionally, soil enzyme activities, particularly those of invertase and urease, have been shown to be sensitive indicators of soil biological fertility and reflect the dynamic influence of N on nutrient transformation processes (Glibert, 2016). Advanced technologies, such as ^15^N isotopic tracing, have enabled precise quantification of N flows within the system—partitioning N uptake by plants, soil residuals, and environmental losses—thereby offering technical insight into NUE variations (Li et al., 2024; Zhong et al., 2025a, 2025b).

Despite these advances, most existing studies remain confined to isolated perspectives, often focusing on either plant uptake or soil transformation alone, and rarely consider the sugarcane-soil system as a tightly coupled, interactive whole. A particularly underexplored dimension is ecological stoichiometry, which offers a theoretical framework to examine how N inputs affect the balance of C, N, and P across plant and soil compartments (Reich and Oleksyn, 2018). The C:N:P ratio is a key indicator of nutrient transformation efficiency, microbial metabolism, and system stability. Without assessing this Nitrogen element, it is difficult to comprehensively understand the synergistic effects of N on the overall system or to construct optimized, ecologically rational N strategies (Stefanson, 1972).

To fill this critical knowledge gap, this study proposes an integrated research framework linking plant performance, N fate and stoichiometric balance, with the hypothesis that medium one-time N application optimizes plant-soil C:N:P balance, thus synergistically boosting sugarcane yield, NUE and soil quality. Combining ^15^N tracing and stoichiometric analysis, it aims to elucidate N’s synergistic regulatory mechanisms in the sugarcane-soil system, laying a scientific foundation for sustainable sugarcane production.

## 2 Materials and methods

### 2.1 Study site and experimental materials

#### 2.1.1 Study site description

The pot experiment was carried out in 2023 within a greenhouse located at the Agricultural Science Research Institute of Tianyang District, Baise City, Guangxi Zhuang Autonomous Region, which was roughly located at 23°40′57″ N, 106°58′58″ E, with an altitude of approximately 120 meters. In Guangxi, the annual outdoor temperature ranges from 10 °C to 36 °C from March to December and annual rainfall ranging between 1100 and 1300 mm, characterized by a southern subtropical monsoon climate. The greenhouse environment was designed to closely mimic local field conditions, featuring natural light and ventilation, with daily temperature variations maintained within ±2 °C of the outdoor ambient range. The experimental site was deliberately chosen due to its representative soil characteristics and climatic conditions, ensuring that the outcomes of this study would be relevant and applicable to sugarcane production practices across Guangxi (Wang and Cai, 2024).

#### 2.1.2 Test soil

The experimental soil used in this study was classified as Alisols (yellow-brown type) following the World Reference Base for Soil Resources 2022 (4th edition, update 2024) (WRB, 2024). It is moderately acidic, with a heavy clay texture, developed fissures and funnel structures, and is characterized by strong leaching, poor nutrient retention, and high spatial heterogeneity. sampled from the 0–20 cm plow layer within the greenhouse. After air-drying under natural conditions, visible gravel and root residues were manually removed, and the soil was subsequently sieved through a 5 mm mesh to prepare it for potting. Key physicochemical properties were determined following established protocols (Bao, 2000). The soil exhibited a pH of 6.71, measured using a potentiometric method (PHS-3C pH meter, Shanghai INESA Scientific Instrument Co., Ltd.). OM content was 52.18 g/kg, assessed via the K dichromate oxidation method. Total N (TN) was measured at 0.40 g/kg using the Kjeldahl method with a K9840 automatic analyzer (Hanon Future Technology Group Co., Ltd.). Available P (AP) reached 58.73 mg/kg, extracted with sodium bicarbonate and quantified via the Mo-Sb colorimetric method. Available K (AK) was determined at 55.05 mg/kg, using ammonium acetate extraction and a flame photometer (FP640, Shanghai INESA) (Cai et al., 2025). Ecological stoichiometric analysis revealed C:N, C:P, and N:P ratios of 24.3, 482.5, and 19.8 (Blair et al., 1989), respectively. These values indicate moderate soil fertility, suitable for supporting sugarcane growth and providing a reliable foundation for assessing the effects of N management strategies (Plenchette et al., 1983).

#### 2.1.3 Test crop

The sugarcane cultivar employed in this study was ‘Guitang 46’, developed by the Sugarcane Research Institute of the Guangxi Academy of Agricultural Sciences. This medium-maturing variety is characterized by a growth cycle of 11 to 12 months and an average sucrose content ranging from 15.2% to 16.5% at maturity. Notably, it exhibits strong resistance to abiotic stresses, including cold tolerance down to 5 °C and the ability to sustain approximately 80% of its typical yield under mild drought conditions. As the predominant cultivar widely cultivated in the sugarcane-producing regions of Baise, ‘Guitang 46’ is highly adapted to the local agroecological environment. Its physiological and agronomic traits ensure the reliability and representativeness of experimental outcomes derived from this study (Elshobary et al., 2025).

#### 2.1.4 Test fertilizers and instruments

The fertilizers applied in this experiment included ^15^N-labeled urea (10.08 atom% ^15^N excess; Shanghai Research Institute of Chemical Industry), conventional urea with 46% N content (Tianjin Zhiyuan Chemical Reagent Co., Ltd.), superphosphate containing 12% P_2_O_5_, and KCl with 60% K_2_O (both supplied by the same manufacturer). All fertilizer materials conformed to agricultural research-grade standards, with purity levels exceeding 99.0% (Högberg, 1997).

All analytical instruments were calibrated in accordance with national standards prior to use. Measurements were conducted using the following equipment: an analytical balance (Model TX224, Shimadzu International Trade, Shanghai; precision: 0.1 mg) for sample weighing; a K9840 automatic Kjeldahl nitrogen analyzer (Hanon) for determining TN; and a Vario EL III elemental analyzer (Elementar, Germany; accuracy ±0.1%) for total C (TC) quantification. Sample drying was performed using an electric blast drying oven (Model 101-3AB, Tianjin Taisite Instrument Co., Ltd.) with a temperature control precision of ±1 °C. For isotope and biochemical analyses, a MAT 253 isotope ratio mass spectrometer (Thermo Fisher Scientific, USA) was used to determine ^15^N abundance, while an ultraviolet spectrophotometer (Model UV-2600, Shimadzu) was employed for measuring enzyme activity and inorganic N concentrations.

### 2.2 Experimental design

A pot experiment was conducted using bottomless plastic buckets (diameter: 0.50 m; height: 0.55 m; volume: 0.43 m³). The 60%–70% field water capacity (FWC) was maintained to promote root and shoot establishment; avoid overwatering to prevent seed rot. Six N treatments were established, each replicated five times, resulting in 30 pots arranged in a randomized complete block design. To better simulate field cultivation conditions, protective rows of sugarcane were planted around the pots to mimic the local microclimate (Wang and Cai, 2024).

The experimental design employed a dual-factor regulatory framework, incorporating N application mode (one-time vs. split) and application rate (low, medium, high) as core variables. This allowed for a systematic assessment of their combined effects on the sugarcane-soil system, with the aim of identifying a N management strategy optimized for both dosage and application method. The average conventional nitrogen application rate in sugarcane field in Guangxi is 20–25 kg/ha, which is the general fertilization level for local production.

The specific N application rates and methods were as follows (**Table 1**): L0 (Control): no N applied; L1 (Low N, split application): 185.85 kg/ha (4.96 g/pot), with 30% applied as base fertilizer (1.49 g/pot), 30% during tillering (1.49 g/pot), and 40% at the elongation stage (1.92 g/pot); L2 (Medium N, split application): 371.70 kg/ha (9.91 g/pot), using the same split ratio as L1; L3 (High N, split application): 557.55 kg/ha (14.87 g/pot), also using the same split ratio; L4 (Medium N, one-time application): 371.70 kg/ha (9.91 g/pot), fully applied as base fertilizer; and L5 (High N, one-time application): 525.00 kg/ha (20.00 g/pot), also fully applied as base fertilizer All treatments received consistent basal applications of P and K: 525 kg/ha superphosphate (equivalent to 14 g/pot) and 450 kg/ha KCl (equivalent to 12 g/pot), which were thoroughly mixed with the soil and base fertilizer prior to planting (Wang and Cai, 2024).

**Table 1:** Nitrogen application treatments in the pot experiment.

Treatment	N rate (kg/ha)	N rate (g/pot)	Application method	Split details (% of total N and g/pot)
L0 (Control)	0	0		
L1 (Low, split)	185.85	4.96	Split (three times)	Basal: 30% (1.49 g); Tillering: 30% (1.49 g); Elongation: 40% (1.92 g)
L2 (Medium, split)	371.7	9.91	Split (three times)	Basal: 30% (2.97 g); Tillering: 30% (2.97 g); Elongation: 40% (3.96 g)
L3 (High, split)	557.55	14.87	Split (three times)	Basal: 30% (4.46 g); Tillering: 30% (4.46 g); Elongation: 40% (5.95 g)
L4 (Medium, one-time)	371.7	9.91	One-time (basal)	100% basal (9.91 g)
L5 (High, one-time)	525	20.00	One-time (basal)	100% basal (20.00 g)

All treatments received the same basal application of P and K: 14 g/pot superphosphate (equivalent to 525 kg/ha) and 12 g/pot KCl (equivalent to 450 kg/ha), thoroughly mixed with soil before planting.

Sugarcane setts (two-bud segments) were planted on April 13, 2023. After seedling emergence, two healthy plants were retained per pot. Pest control was carried out during the growth period by spraying a 2000-fold dilution of Fengman to control red spider mites and a 1500-fold dilution of Dasajie to manage pink mealybugs, with all pesticide applications performed in the early morning to prevent phytotoxicity (Wang and Cai, 2024).

### 2.3 Sample collection and preparation

Samples were collected on December 26, 2023, with a total growth period of 258 days (from April 13 to December 26, 2023), corresponding to the mature stage of sugarcane growth.(Each plant was separated into four distinct organs: root, stem, new leaf (defined as the uppermost three fully expanded leaves), and old leaf (lower leaves exhibiting approximately 50% chlorosis). The fresh weight of each organ was recorded immediately after harvest. To deactivate enzymatic activity, samples were heated at 105 °C for 30 min, followed by oven-drying at 75 °C until a constant weight was achieved. Dried samples were then ground and passed through a 100-mesh sieve for subsequent determination of N content, TC content, and ^15^N abundance (Cai et al., 2024). Soil samples were collected using a soil auger from three depth intervals: surface (0–15 cm), middle (15–30 cm), and bottom (30–45 cm) layers within each pot. After removing visible plant debris and gravel, the samples were air-dried at ambient temperature for seven days, ground, and sequentially sieved through 20-mesh and 100-mesh nylon screens. A quartering method was employed to obtain representative subsamples for the analysis of soil physicochemical characteristics, enzyme activity profiles, and ecological stoichiometric ratios (Jacobsen et al., 1992).

### 2.4 Determination items and methods

#### 2.4.1 Sugarcane plant indicators

Dry matter weight was measured by weighing the oven-dried samples using an analytical balance (Model TX224, Shimadzu) (Wang and Cai, 2024). For elemental analysis, plant samples were digested in a mixture of H_2_SO_4_ and H_2_O_2_ (volume ratio 5:1) at 380 °C for 2 h. TN content was determined using an automatic Kjeldahl nitrogen analyzer (Model K9840, Hanon), TP via the V-Mo yellow colorimetric method using a UV-2600 spectrophotometer, and TK by flame photometry (Model FP640) (de Castro et al., 2019). To determine ^15^N abundance, 20 mg of finely ground sample was sent to Shandong Standard Testing Technology Co., Ltd. and analyzed using an isotope ratio mass spectrometer (Model MAT 253, Thermo Fisher Scientific) (Wang and Cai, 2024). TC content was quantified with an elemental analyzer (Model Vario EL III, Elementar, Germany), offering a measurement precision of ±0.1%.

#### 2.4.2 Soil indicators

Soil pH was determined using the potentiometric method with a soil-to-water ratio of 1:2.5, measured by a PHS-3C pH meter. OM content was quantified using the K dichromate volumetric method under external heating conditions (Bao, 2000). TN was measured via the Kjeldahl method using a K9840 automatic nitrogen analyzer, while TP was determined following H_2_SO_4_–HClO_4_ digestion and subsequent analysis by the V-Mo yellow spectrophotometric method. NH_4_^+^-N and NO_3_^−^-N were extracted with 2 mol/L KCl solution at a soil-to-solution ratio of 1:10, shaken at 200 rpm for 30 min, and quantified using a UV-2600 ultraviolet spectrophotometer (Bao, 2000). Soil enzyme activities were assessed as follows: invertase and cellulase activities were measured using the 3,5-dinitrosalicylic acid colorimetric method after incubation at 37 °C for 24 h and absorbance reading at 540 nm; urease activity was determined via the phenol-sodium hypochlorite colorimetric method after 24-h incubation at 37 °C and measured at 578 nm; acid phosphatase activity was measured using the sodium phenylphosphate colorimetric method after 1-h incubation at 37 °C with detection at 400 nm; and catalase activity was evaluated via K permanganate titration (Bao, 2000).

#### 2.4.3 Calculation of N fate

Nitrogen fate parameters were calculated based on the method described by Wang and Cai (2024). The proportion of N derived from fertilizer (Ndff, %) was calculated using the Equation 1. N uptake by each plant organ (g/plant) was obtained by multiplying the dry matter weight of the organ by its TN content. The ^15^N recovery rate (%) was calculated as the ratio of total ^15^N accumulation in the plant to the total ^15^N input from fertilizer, multiplied by 100. The ^15^N residual rate (%) was determined as the proportion of ^15^N remaining in the soil relative to the ^15^N input from fertilizer. The ^15^N loss rate (%) was derived by subtracting the ^15^N recovery and residual rates from 1 (i.e., ^15^N loss rate = 1 − ^15^N recovery rate − ^15^N residual rate).

(1) N 15 abndance =N 15 abundance in the sample− naturalN 15 abundance (0.3663%)N 15 abundance in labeled fertilizer − naturalN 15 abundance  × 100

#### 2.4.4 Calculation of ecological stoichiometric ratios

Ecological stoichiometric ratios were calculated based on the measured contents of TC, TN, and TP (Franco et al., 2015). The C:N ratio was determined as C:N = TC/TN; C:P = TC/TP; N:P = TN/TP.

### 2.4 Data processing and statistical analysis

All data were organized and processed using Microsoft Excel 2019, and graphical illustrations were generated with Origin 2022. Statistical analyses were carried out using IBM SPSS Statistics 22.0. One-way analysis of variance (ANOVA) was performed to assess significant differences among treatment groups, followed by Duncan’s multiple range test for *post-hoc* comparisons, with statistical significance set at p < 0.05. In addition, correlation analysis (Pearson) was conducted to examine the relationships between soil physicochemical properties and sugarcane growth parameters (Wang and Cai, 2024).

## 3 Results and analysis

The effects of varying N application regimes—both in terms of application rate and method—on the sugarcane-soil system were statistically significant. Consistent regulatory trends were observed across multiple core indicators, including sugarcane growth performance, N uptake and distribution, soil physicochemical properties, and ecological stoichiometric ratios. Among all treatments, the medium-rate one-time application (L4) demonstrated superior performance across these parameters and was determined to the most effective strategy, as elaborated in the following results.

### 3.1 Effects of N treatments on sugarcane dry matter accumulation, N uptake, and distribution

**Figure 1:**
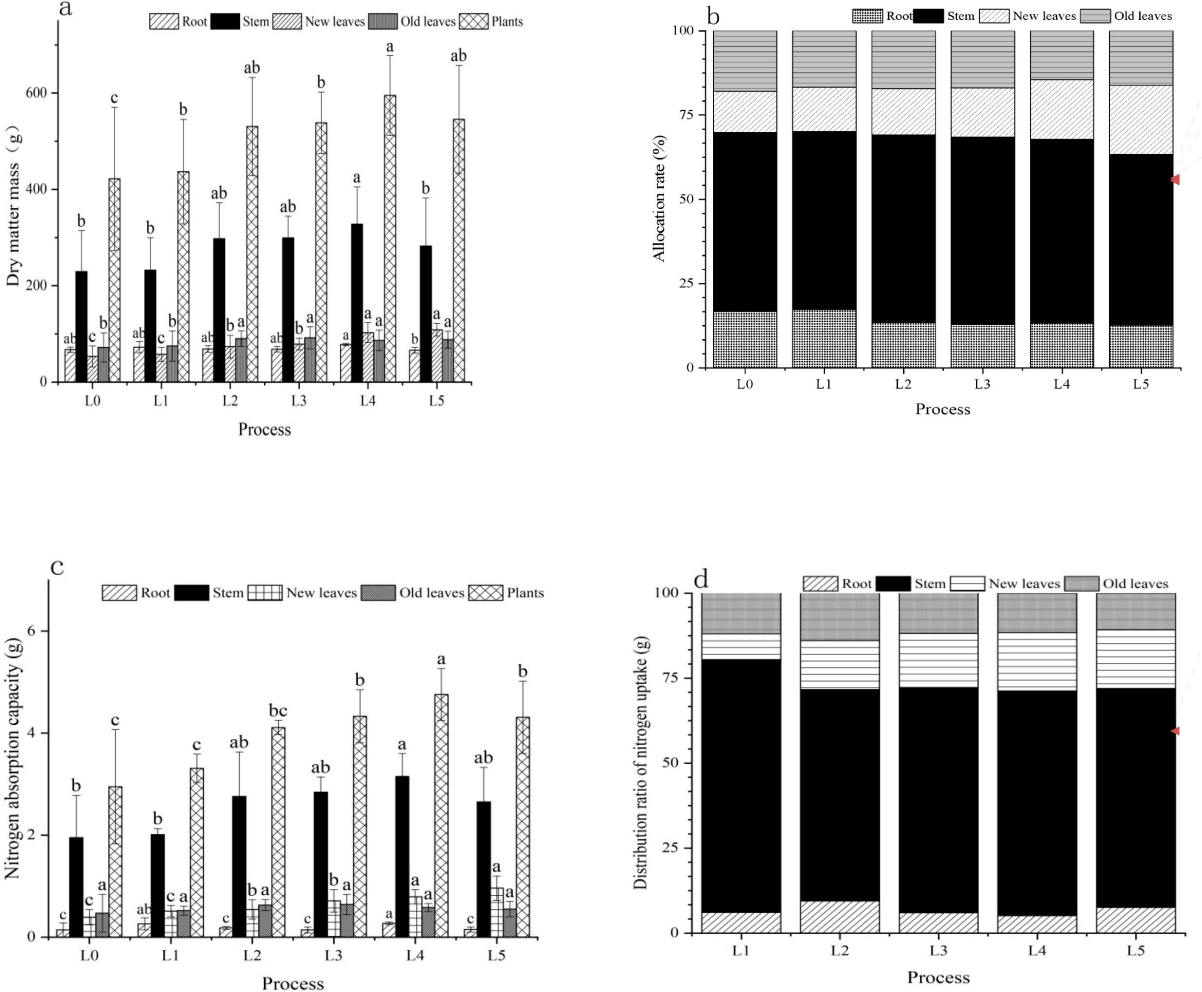
Effects of different nitrogen application regimes on sugarcane growth and nitrogen uptake. **(A)** Dry matter accumulation, **(B)** distribution, **(C)** Nitrogen uptake and **(D)** distribution of sugarcane under different treatments under six N treatments (L0, no N applied; L1, low split; L2, medium split; L3, high split; L4, medium one-time; L5, high one-time). Different lowercase letters indicate significant differences among treatments (one-way ANOVA: F_5_,_24_ = 12.76, P < 0.05, df_1_ = 5, df_2_ = 24).

All N treatments significantly enhanced sugarcane dry matter accumulation compared to the no-N control (L0) (**Table 2****;** **Figure 1**), with clear differences observed in both organ-specific allocation and total biomass (**Table 2****;** **Figure 1**). Across all treatments, the dry matter distribution among sugarcane organs followed a consistent trend: stem > new leaves > old leaves > roots (P < 0.05) (**Table 2****;** **Figure 1B**). The L4 treatment yielded the highest stem dry matter (327.75 g) and total plant dry matter (594.96 g) (**Table 2**), representing increases of 42.87% and 41%, respectively, compared to L0 (229.40 g and 421.96 g) (**Table 2**). Additionally, plants under L4 exhibited the greatest height (161.9 cm/plant) and fresh weight (1.08 kg/plant) (**Table 2**), confirming its advantage in promoting yield-related traits.

**Table 2:** Distribution of ^15^N in sugarcane-soil system under different N application treatments.

Process	^15^N input (g/pot)	Sugarcane ^15^N amount (g/pot)	Recovery rate (%)	Soil ^15^N residual amount (g/pot)	Residual rate (%)	Loss rate (%)
L1	1.17	0.43 d	37.01	0.57d	48.49	14.50
L2	2.48	0.79 c	31.94	1.05 b	42.22	25.84
L3	3.72	0.93 bc	24.90	1.19 b	31.96	43.14
L4	2.48	1.01 a	40.78	0.96 c	38.56	20.66
L5	5.00	1.20 b	24.06	2.57 a	51.36	24.58

Values within the same column followed by different lowercase letters are significantly different at P < 0.05.

Treatment abbreviations: L0, no N input (control); L1, low N split application; L2, medium N split application; L3, high N split application; L4, medium one−time N application; L5, high one−time N application.

**Figure 2:**
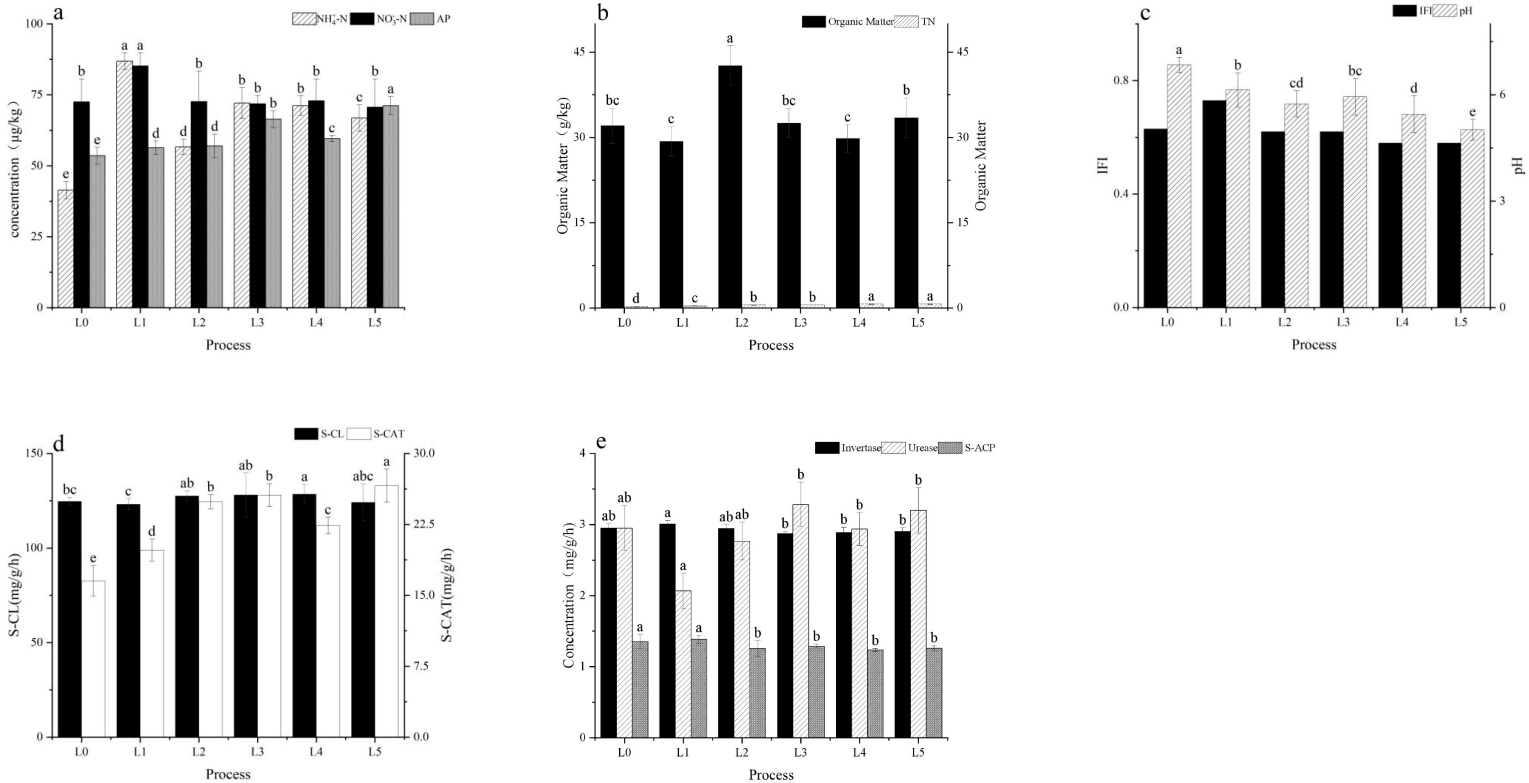
Effects of different N treatments on soil nutrient contents and enzyme activities: **(A)** Nitrate nitrogen (NO_3_^−^-N), ammonium nitrogen (NH_4_^+^-N), and available phosphorus (AP); **(B)** Soil organic matter (OM) and total nitrogen (TN); **(C)** Soil potential of hydrogen (pH) and integrated fertility index (IFI); **(D)** cellulase, catalase, and acid phosphatase activities; **(E)** invertase and urease activities under different treatments under six N treatments (L0, no N applied; L1, low split; L2, medium split; L3, high split; L4, medium one-time; L5, high one-time). Different lowercase letters indicate significant differences among treatments (one-way ANOVA: F_5_,_24_ = 12.76, P < 0.05, df_1_ = 5, df_2_ = 24).

In terms of N uptake, L4 also exhibited optimal performance (**Table 3****,** **Figure 1**). The stem N uptake under L4 reached 3.15 g/plant (**Table 3**), which was 61.54% higher than L0 (1.95 g/plant) (**Table 3**), while total N uptake reached 4.79 g/plant (**Table 3**)—62.37% higher than L0 (2.95 g) (**Table 3**). Moreover, the N contents in new leaves (1.71 g/kg), stems (0.16 g/kg), and old leaves (0.15 g/kg) under L4 were more balanced compared to other treatments (**Table 3**), indicating more coordinated internal N allocation. The proportion of Ndff further demonstrated enhanced NUE under L4, with stem and new leaf Ndff values of 27.25% and 27.35%, respectively (**Table 3**), significantly exceeding those of the split-application treatments (L1: 21.12%, L2: 23.58%, L3: 22.89%) (**Table 3**). Although the high-rate one-time application (L5) showed the highest stem Ndff (29.07%) (**Table 3**), its total N uptake was lower than L4. Considering both yield and nitrogen use efficiency, L4 was identified as the optimal treatment, whereas L5, despite exhibiting the highest nitrogen use efficiency, did not achieve the optimal yield. Furthermore, L4 maintained sufficient levels of soil inorganic N, with 86.90 mg/kg of NH_4_^+^-N and 255.53 mg/kg of NO_3_^−^-N (**Figure 2A**), thereby ensuring sustained N availability throughout the growth period.

**Table 3:** Path analysis of the effects of soil physicochemical properties and enzyme activities on sugarcane yield.

Action factor	Direct effects	Indirect effects																	
		→X1	→X2	→X3	→X4	→X5	→X6	→X7	→X8	→X9	→X10	→X11	→X12	→X13	→X14	→X15	→X16	→X17	→X18
X1	0.73		0.12	-0.25	0.13	0.14	0.14	0.08	-0.25	-0.31	0.08	0.19	-0.18	-0.06	0.10	-0.15	0.06	0.16	-0.13
X2	-0.94	-0.16		0.14	-0.44	-0.55	-0.53	-0.28	0.14	0.04	-0.50	-0.26	-0.34	0.51	0.43	-0.17	0.42	-0.29	0.38
X3	0.00	0.00		0.00	0.00	0.00	0.00	0.00	0.00	0.00	0.00	0.00	0.00	0.00	0.00	0.00	0.00	0.00	0.00
X4	-0.84	-0.15	-0.40	0.51		-0.79	-0.78	-0.42	0.51	0.14	-0.63	-0.69	-0.54	0.51	0.44	-0.13	0.43	-0.60	0.66
X5	0.66	0.13	0.39	-0.37	0.62		0.64	0.32	-0.37	-0.16	0.50	0.54	0.42	-0.40	-0.36	0.10	-0.41	0.50	-0.55
X6	1.59	0.31	0.89	-0.83	1.47	1.53		0.81	-0.83	-0.45	1.26	1.18	0.98	-1.04	-0.83	0.09	-0.88	1.17	-1.23
X7	-0.16	-0.02	-0.05	0.09	-0.08	-0.08	-0.08		0.09	-0.04	-0.07	-0.08	-0.06	0.06	0.04	-0.05	0.04	-0.05	0.05
X8	0.43	-0.15	-0.07	0.43	-0.26	-0.24	-0.23	-0.25		-0.08	-0.15	-0.30	-0.15	0.08	0.11	-0.03	0.12	-0.13	0.21
X9	1.08	-0.47	-0.04	-0.20	-0.18	-0.26	-0.30	0.30	-0.20		-0.28	-.0	0.06	0.23	-.0	0.22	0.04	-0.38	0.16
X10	0.03	-.0	0.02	-0.01	0.02	0.02	0.03	0.01	-0.01	-0.01		0.02	0.01	-0.01	-0.01	-.0	-0.01	0.02	-0.02
X11	-0.74	-0.19	-0.21	0.51	-0.60	-0.60	-0.55	-0.37	0.51	-.0	-0.39		-0.39	0.24	0.33	-0.10	0.27	-0.47	0.58
X12	0.37	-0.09	0.13	-0.13	0.24	0.23	0.23	0.13	-0.13	0.02	0.16	0.20		-0.13	-0.18	0.15	-0.09	0.19	-0.22
X13	-0.21	0.02	0.11	-0.04	0.12	0.13	0.14	0.08	-0.04	-0.04	0.09	0.07	0.08		-0.12	0.06	-0.09	0.10	-0.11
X14	-0.04	-0.01	0.02	-0.01	0.02	0.02	0.02	0.01	-0.01	-.0	0.02	0.02	0.02	-0.03		0.02	-0.01	0.01	-0.02
X15	0.12	-0.03	0.02	-0.01	0.02	0.02	0.01	0.04	-0.01	0.02	-.0	0.02	0.05	-0.03	-0.04		-0.02	0.02	-0.03
X16	-0.30	-0.03	0.14	-0.08	0.15	0.19	0.17	0.07	-0.08	-0.01	0.13	0.11	0.08	-0.13	-0.09	0.05		0.13	-0.19
X17	-0.09	-0.02	-0.03	0.03	-0.06	-0.07	-0.07	-0.03	0.03	0.03	-0.05	-0.06	-0.05	0.04	0.02	-0.01	0.04		0.07
X18	0.10	-0.02	-0.04	0.05	-0.08	-0.09	-0.08	-0.03	0.05	0.02	-0.05	-0.08	-0.06	0.06	0.04	-0.02	0.07	-0.08	

Coefficient of determination (R²) = 0.639, residual path coefficient = 0.601, df = 126.

Treatment abbreviations: L0, no N input (control); L1, low N split application; L2, medium N split application; L3, high N split application; L4, medium one−time N application; L5, high one−time N application.

### 3.2 Effects of N treatments on soil physicochemical properties, fertility, and enzyme activities

N treatments significantly altered soil nutrient status, fertility, and enzyme activity levels, with the L4 treatment striking an optimal balance between nutrient supply and biological function (**Figure 2**). In terms of soil properties, L4 exhibited the highest level of OM at 63.97 g/kg (**Figure 2B**), which was 41.50% greater than that of L0 (45.20 g/kg) and 22.7% higher than L5 (52.10 g/kg) (**Figure 2B**). While its TN (0.71 g/kg) was comparable to that of L5 (0.72 g/kg) (**Figure 2B**), L4 showed significantly higher available N levels (sum of NH_4_^+^-N and NO_3_^−^-N: 342.43 mg/kg) than all other treatments (**Figure 2A**), indicating more effective N release and reduced risk of N immobilization due to excessive OM accumulation (Conen et al., 2008).

Soil fertility, assessed using the integrated fertility index (IFI), revealed that L1 had the highest IFI value (0.73) (**Figure 2C**); however, its ^15^N recovery rate (37.01%) was lower than that of L4 (40.78%) (**Table 2**), suggesting a disconnect between measured fertility and actual NUE. Although L4 and L5 shared the same IFI score (0.58) (**Figure 2B**), L5 had a notably higher ^15^N loss rate (24.58%) compared to L4 (20.66%) (**Table 2**), indicating reduced sustainability in fertility retention and nutrient utilization (Baite et al., 2025).

Regarding soil enzyme activities—key indicators of soil biological functioning—L4 maintained a balanced enzymatic profile. Invertase (257.00 mg/g/h) and urease (26.61 mg/g/h) activities under L4 were significantly higher than those under the control (L0: 189.50 mg/g/h and 17.82 mg/g/h, respectively) (**Figure 2E**), facilitating enhanced C and N cycling. Meanwhile, cellulase (1.20 mg/g/h) and catalase (2.80 mg/g/h) activities in L4 were comparable to those of L2 (1.18 mg/g/h and 2.75 mg/g/h) (**Figure 2D**), suggesting that L4 avoided the inhibitory effects on enzyme function often associated with excessive N input. In contrast, although L5 exhibited elevated urease activity (28.30 mg/g/h) (**Figure 2E**), its low acid phosphatase level (2.10 mg/kg) (**Figure 2D**) indicated an imbalance in nutrient transformation processes.

### 3.3 Effects of n treatments on n fate and balance in sugarcane-soil system

**Figure 3:**
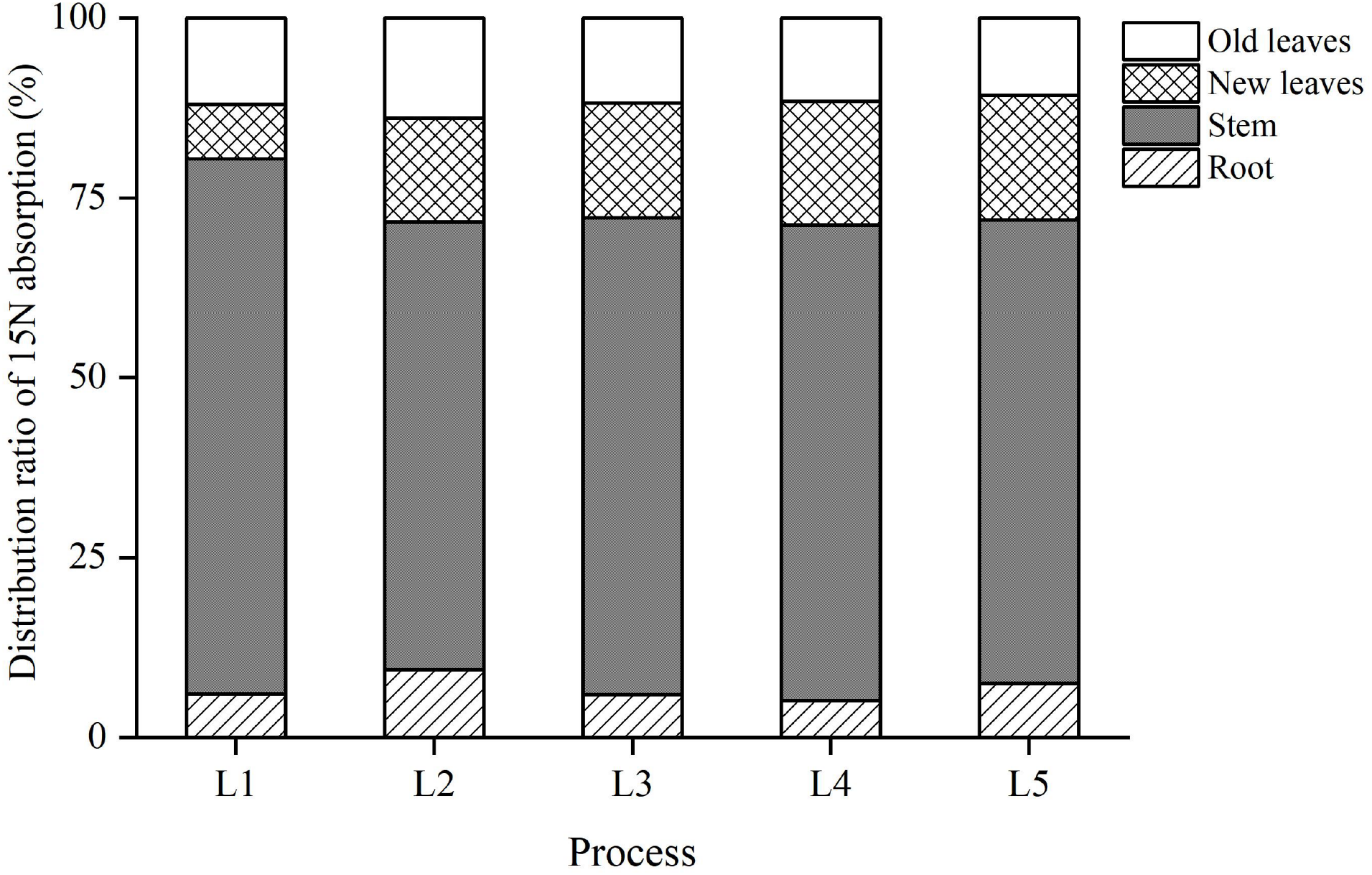
Distribution ratio of ^15^N uptake among sugarcane organs under six N treatments (L0, no N applied; L1, low split; L2, medium split; L3, high split; L4, medium one-time; L5, high one-time).

^15^N tracing revealed significant differences in N distribution across treatments, with L4 demonstrating the most effective coordination of N allocation among plant, soil, and environmental compartments (**Table 2****;** **Figure 3**). Specifically, L4 achieved the highest ^15^N recovery rate at 40.78% (**Table 2**), which was 3.77 percentage points higher than that of L1 (37.01%) and 15.88 points higher than L3 (24.90%) (**Table 2**). Its soil ^15^N residual rate was 38.56% (**Table 2**), markedly lower than L5 (51.36%) (**Table 2**), thereby reducing N redundancy and the associated risk of leaching. Furthermore, L4 exhibited the lowest ^15^N loss rate (20.66%) among all N-fertilized treatments (**Table 2**), which was 22.48 percentage points lower than that observed in L3 (43.14%) (**Table 2**), indicating superior N retention and environmental performance.

Correlation analysis further supported the regulatory advantage of L4. A significant positive correlation was observed between soil TN and new leaf N content (r = 0.68, p < 0.01), while the soil C:N ratio showed a significant negative correlation with stem C:N ratio (r = −0.52, p < 0.05). These relationships suggest that L4 facilitated a well-coordinated cycle among N supply from the soil, its uptake by sugarcane, and the decomposition of OM—forming a dynamic “supply-demand-transformation” feedback loop (Li et al., 2024).

### 3.4 Effects of n treatments on ecological stoichiometric characteristics of sugarcane organs and soil

**Figure 4:**
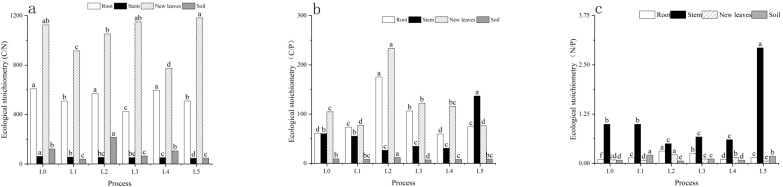
Ecological stoichiometric ratios of **(A)** C:N, **(B)** C:P, and **(C)** N:P in sugarcane and soil under six N treatments (L0, no N applied; L1, low split; L2, medium split; L3, high split; L4, medium one-time; L5, high one-time). Different lowercase letters indicate significant differences among treatments (one-way ANOVA: F_5_,_24_ = 12.76, P < 0.05, df_1_ = 5, df_2_ = 24).

N treatments significantly influenced C:N:P ratios—key indicators of nutrient balance—at both the plant and soil levels, with L4 demonstrating the most favorable stoichiometric adjustments (**Figure 4**). In sugarcane organs, the stem C:N ratio under L4 (28.5) (**Figure 4A**) was lower than that of L5 (35.2) (**Figure 4A**) but higher than L1 (24.3) (**Figure 4A**), suggesting improved N conversion efficiency into structural C, which aligns with the highest stem dry matter observed under L4. The N:P ratio in new leaves (14.2) (**Figure 4C**) fell within the optimal physiological range for nutrient homeostasis (10~16), avoiding N limitation (L0: 8.9) (**Figure 4C**) or P limitation (L3: 18.7) (**Figure 4C**). The root C:P ratio under L4 was 325.6 (**Figure 4B**)—the lowest among all treatments—indicating enhanced P uptake, consistent with L4’s elevated level of available soil P (28.90 mg/kg) (**Figure 2A**) (Reich and Oleksyn, 2018).

At the soil level, L4 also exhibited optimal stoichiometry. Its soil C:N ratio (18.7) (**Figure 4A**) was close to the ideal threshold for microbial decomposition (approximately 25), in contrast to the lower C:N ratio in L5 (15.2) (**Figure 4A**), which may indicate excessive OM breakdown, and the higher C:N in L0 (24.3) (**Figure 4A**), associated with reduced decomposition activity. This pattern corresponded with L4’s elevated invertase activity and minimized N loss rate. Furthermore, L4’s balanced soil N:P ratio (5.8) (**Figure 4C**) avoided two core TP negative pathways: P surplus-induced fixation and inactivation (L1, 3.2) and P limitation-induced nutrient cycling restriction (L3, 8.9) (**Figure 4c**); its lowest soil C:P ratio (325.6) (**Figure 4b**) further confirmed enhanced P activation and bioavailability.

### 3.5 Relationships between soil nutrients, ecological stoichiometry, and yield-driving mechanisms

**Figure 5:**
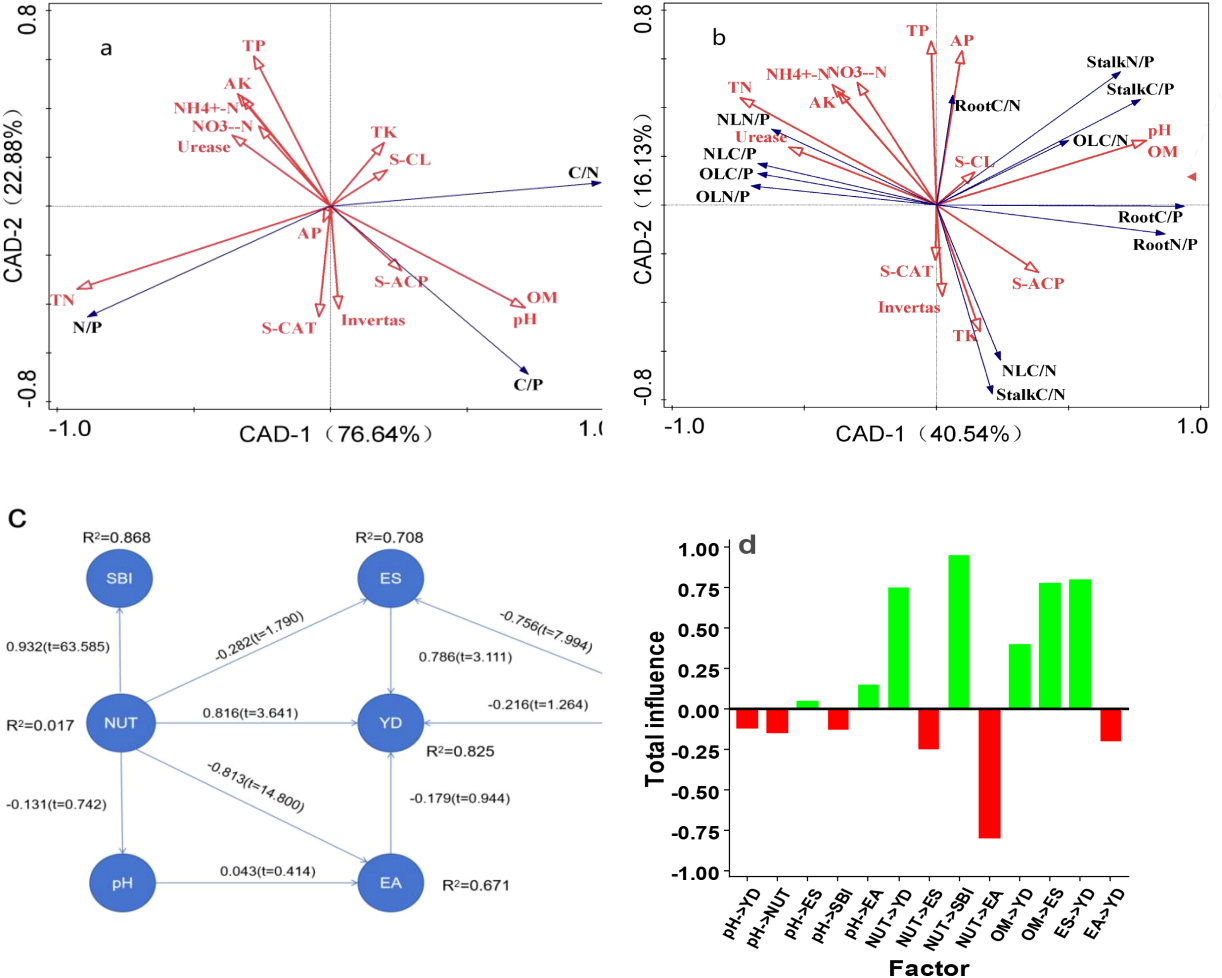
RDA: multivariate analyses of ecological stoichiometry and yield-driving mechanisms under six N treatments (L0: no N applied; L1: low split; L2: medium split; L3: high split; L4: medium one-time; L5: high one-time): **(A)** RDA of soil ecological stoichiometry; **(B)** RDA of plant ecological stoichiometry; **(C)** SEM; **(D)** Path coefficients of key influencing factors. Different lowercase letters indicate significant differences among treatments (P < 0.05, df = 5, 24.).

Redundancy analysis (RDA) revealed strong quantitative relationships between soil nutrients and ecological stoichiometric parameters (**Figure 5**). For soil, the cumulative explanatory power of nutrients for stoichiometric variation reached 99.52% (**Figure 5A**), with PC1 and PC2 accounting for 76.64% and 22.88% of the variation, respectively (**Figure 5A**). Among the influencing factors, soil TK and cellulase activity emerged as the primary drivers, both showing significant positive correlations with the soil C:N ratio (p < 0.01) (**Figure 5A**). In the sugarcane system, the cumulative explanatory rate was 54.01% (PC1: 37.64%; PC2: 16.37%) (**Figure 5B**). In this study, OM and AP were the dominant contributors to stoichiometry in old leaves and roots, as indicated by their positive correlation with the corresponding C:N ratios (p < 0.01) (**Figure 5B**), while TK was the main driver for stoichiometric variation in new leaves and stems (also positively correlated with C:N ratios, p < 0.01) (**Figure 5B**).

Structural equation modeling (SEM) further demonstrated that ecological stoichiometry exerted the strongest direct influence on sugarcane yield, while soil nutrients and OM contributed significant indirect effects. Soil nutrients were positively correlated with base ion concentrations, thereby enhancing nutrient supply capacity, but were negatively correlated with enzyme activities—suggesting that excessive enzyme activity may suppress nutrient accumulation. Path analysis identified AK as the most influential positive driver of yield, with a direct path coefficient of 1.59, followed by OM (1.08), whereas TP exhibited the most substantial negative effect (−0.94, **Table 3**). Notably, AK also exerted the strongest indirect influence on soil NH_4_^+^-N and NO_3_^−^-N levels among all measured variables, underscoring its pivotal role in improving N availability and supporting sugarcane yield formation (Palacios-Linares and Guzman, 2024).

## 4 Discussion

This study investigated the regulatory effects of N application rate and method on the sugarcane-soil system by integrating ^15^N tracing techniques with ecological stoichiometric analysis. The research aimed to address the central challenge of reconciling high crop yield, efficient N utilization, and improved soil quality in sugarcane production. The findings clearly demonstrated that the medium-rate one-time N application (L4, conferred integrated improvement across sugarcane growth, N cycling, and soil ecological functionality. The following discussion explores the underlying mechanisms driving these outcomes and examines the differences in responses among the various N treatments.

### 4.1 Synergistic regulatory mechanism of medium N one-time application: coupling of growth, N use, and stoichiometry

The medium-rate one-time N application (L4) exhibited the most comprehensive performance across all treatments, with stem dry matter (327.75 g) (**Table 2**) and total plant N uptake (4.79 g/plant) (**Table 2**) increasing by 42.87% and 62.37%, respectively, compared to the L0. This superior stem dry matter accumulation reflects the species’ characteristic strategy of prioritizing photosynthate storage in the stem (Zhong et al., 2025a). This was accompanied by a high ^15^N recovery rate (40.78%) (**Table 2**) and a low loss rate (20.66%) (**Table 2**), indicating that L4 effectively synchronized soil N supply with the plant’s nutritional demand. From the perspective of N supply dynamics, the one-time basal application minimized the risks of N volatilization or leaching typically associated with split applications—especially during the tillering and elongation stages, where fertilizer remains exposed at the soil surface for extended periods (Li et al., 2024). This finding is consistent with Li et al. (2024), who reported that one-time N fertilization can increase sugarcane ^15^N recovery by 15%~20% relative to split application, due to reduced N turnover frequency in the soil and improved retention.

Ecological stoichiometry further elucidated the mechanisms underlying this synergistic effect. L4 optimized the C:N:P ratios in both sugarcane tissues and soil. The stem C:N ratio (28.5) under L4 reflected a balanced conversion efficiency of N into structural C, in line with its highest stem biomass accumulation. Meanwhile, the N:P ratio in new leaves (14.2) fell within the optimal physiological range for plant nutrient homeostasis (10~16) (Reich and Oleksyn, 2018), thereby avoiding both N limitation (L0: N:P = 8.9) and P limitation (as observed in L3, N:P = 18.7), both of which can impair photosynthetic capacity. At the soil level, the C:N ratio (18.7) approached the optimal threshold (~25) for microbial decomposition, a balance that was mirrored by L4’s moderate invertase (257.00 mg/g/h) and urease (26.61 mg/g/h) activities. This alignment ensured that the rates of OM decomposition and N mineralization were tightly coupled to sugarcane N demand, forming a closed-loop system of “microbial decomposition - soil N supply - plant uptake.” These findings validate the theoretical proposition that “plant-soil C:N:P alignment is central to nutrient use efficiency” (Reich and Oleksyn, 2018).

Additionally, L4 significantly enhanced soil OM (63.97 g/kg) and available N (NH_4_^+^-N: 86.90 mg/kg; NO_3_^−^-N: 255.53 mg/kg), ensuring a sustained nutrient supply for sugarcane growth while maintaining soil structural stability. This result supports the findings of Cai et al. (2024), who emphasized that appropriate N input can stimulate enzyme synthesis, enhance microbial activity, and accelerate nutrient cycling, all without degrading soil fertility.

### 4.2 Key drivers of differential treatment effects: interaction between N rate and application method

The significant variation in outcomes among N treatments was primarily attributed to the interaction between N application rate and method. Although the low-rate split application (L1) achieved the highest IFI (0.73), its new leaf N:P ratio (8.9) fell below the physiological homeostasis threshold, indicating severe N limitation. This nutrient imbalance resulted in a total plant dry matter accumulation of only 437.00 g, substantially lower than that of L4 (594.96 g). Additionally, L1’s soil C:N ratio (21.5) exceeded that of L4, suggesting slower OM decomposition and an insufficient N release rate to meet the crop’s peak N demand during the elongation stage (Lingle, 1999) who reported a significant positive correlation between N uptake and dry matter accumulation under low N conditions, confirming that N deficiency was the primary limiting factor in L1.

In contrast, the high-rate split application (L3) exhibited the highest ^15^N loss rate (43.14%), driven by two key mechanisms. First, the increased frequency of N applications under split regimes intensified the exposure of N to leaching (i.e., NO_3_^−^) and denitrification (i.e., N_2_O) pathways (Zhong et al., 2025b). Second, excessive N input disrupted soil N:P stoichiometry (N:P = 8.9), resulting in P limitation that impeded root development and consequently reduced N uptake capacity. Moreover, L3 led to acidification of the soil (pH = 5.8), which significantly inhibited acid phosphatase activity (only 2.1 mg/kg), further constraining P mobilization and reinforcing a “N excess-P deficiency” imbalance (Kurtz and Hassett, 1957). Similarly, the high-rate one-time application (L5) induced the highest soil ^15^N residual rate (51.36%), as the applied N dose (35.00 kg/666.67 m²) exceeded both the soil colloidal adsorption capacity and microbial immobilization potential, thereby suppressing root absorption capacity (Otto et al., 2019). The resulting soil C:N ratio (15.2) was notably low, accelerating OM mineralization and promoting N loss via leaching before plant uptake could occur, as reflected in a loss rate of 24.58%.

In contrast, L4—through the combination of an appropriate application rate and a one-time basal application strategy—successfully avoided the pitfalls observed in other treatments. It alleviated N limitation (L1), minimized N loss (L3), and prevented N redundancy (L5), ultimately making it the most balanced and effective treatment for enhancing yield while maintaining soil and environmental integrity.

### 4.3 N source characteristics of sugarcane and practical implications for soil fertility improvement

^15^N tracing results revealed two key findings: first, over 70% of the N absorbed by sugarcane was derived from the soil; second, the stem, the primary N allocation organ of sugarcane, had the highest Ndff value (27.25%) across all plant tissues. This finding underscores that soil inherent fertility is the fundamental source of N for sugarcane, and that optimal N management must strike a balance between “immediate fertilization” and “long-term soil fertility enhancement” (Li et al., 2024). While L4 demonstrated clear short-term synergistic benefits, its long-term sustainability hinges on improving the soil’s capacity to store and transform N.

Based on the significant negative correlation observed between soil C:N ratio and sugarcane stem C:N ratio (r = −0.52, p < 0.05), two soil fertility improvement strategies are proposed. First, straw incorporation is recommended to increase soil organic C levels and adjust the C:N ratio toward the optimal microbial decomposition range (20~25), thereby moderating N mineralization rates and prolonging N availability throughout the crop growth cycle. Second, integrated application of organic and inorganic fertilizers can expand the soil N reservoir and improve nutrient buffering capacity. This aligns with the findings of Li et al. (2025), who reported that such combined fertilization strategies in tropical sugarcane systems enhanced soil N storage by 30%~40% and significantly improved NUE.

Furthermore, the significant positive correlation between soil available N (NH_4_^+^-N and NO_3_^−^-N) and TN content in sugarcane new leaves (r = 0.68, p < 0.01) suggests a practical approach for precision fertilization. Real-time monitoring of soil available N could inform dynamic topdressing strategies—for instance, applying supplemental N when available N falls below a threshold (e.g., 150 mg/kg)—thereby further enhancing NUE and crop performance in the field.

This pot experiment under controlled conditions cannot fully mimic field environments. Future field trials will validate the optimal N regime, and long-term studies will explore its impacts on soil microbial communities and N-cycling functional genes for sustainable sugarcane production.

## 4 Conclusions

This study aimed to identify an optimized nitrogen (N) management strategy that synergistically improves sugarcane yield, N use efficiency (NUE), soil quality, and nutrient balance in the sugarcane–soil system, using ^15^N tracing and ecological stoichiometry. Six N application regimes were tested on sugarcane variety Guitang 46 in a karst region of Guangxi. The medium N one−time basal application (371.7 kg N ha^−1^) was confirmed as the optimal regime. It significantly increased stem dry matter and total plant N uptake by 42.87% and 62.37% relative to the control, achieved the highest ^15^N recovery rate (40.78%), and minimized N loss (20.66%). This treatment optimized the C:N:P stoichiometry of both sugarcane tissues and soil, matching ideal thresholds for plant physiology and microbial decomposition. It also enhanced soil organic matter, available N, and key enzyme activities, sustaining efficient nutrient cycling. Over 70% of sugarcane N uptake originated from the soil, highlighting the importance of inherent soil fertility. These findings provide a scientific basis for reducing N fertilizer input while maintaining high and sustainable sugarcane production in subtropical karst areas.

## Data Availability

The raw data supporting the conclusions of this article will be made available by the authors, without undue reservation.
